# A New and Simple Risk Predictor of Contrast-Induced Nephropathy in Patients Undergoing Primary Percutaneous Coronary Intervention: TIMI Risk Index

**DOI:** 10.1155/2018/5908215

**Published:** 2018-09-26

**Authors:** Ahmet Kaya, Ahmet Karataş, Yasemin Kaya, Harun Düğeroğlu, Seçkin Dereli, Adil Bayramoğlu

**Affiliations:** ^1^Department of Cardiology, Ordu University Medical School, Ordu, Turkey; ^2^Department of Nephrology, Ordu University Medical School, Ordu, Turkey; ^3^Department of Internal Medicine, Ordu University Medical School, Ordu, Turkey; ^4^Department of Cardiology, Ordu State Hospital, Ordu, Turkey

## Abstract

**Background:**

The thrombolysis in myocardial infarction risk index (TRI) was developed to estimate prognosis at the initial contact of the healthcare provider in coronary artery disease patients without laboratory parameters. In this study, we aimed to investigate the relationship of the baseline TRI and contrast-induced nephropathy (CIN) in patients with ST-elevation myocardial infarction (STEMI).

**Methods:**

A total of 963 consecutive STEMI diagnosed patients who underwent primary percutaneous intervention were included in the study. TRI was calculated using the formula “heart rate × (age/10) 2/SBP” on admission. CIN was defined as an increase in serum creatinine concentration ≥25%, 48 hours later over the baseline.

**Results:**

Of the total of 963 patients, CIN was observed in 13% (*n*=128). TRI was significantly higher in the CIN (+) group compared with the CIN (−) group (32.9 ± 18.8 vs 19.9 ± 9.9, *P* < 0.001). There was a stronger correlation between CIN and age, diastolic blood pressure, heart rate, Killip class, left ventricular ejection fraction, amount of contrast media, and diabetes mellitus. The amount of contrast media (OR 1.010, 95% CI 1.007–1.012, *P* < 0.001) and TRI (OR 1.047, 95% CI 1.020–1.075, *P*=001) were independent predictors of CIN. The best threshold TRI for predicting CIN was ≥25.8, with a 67.1% sensitivity and 80.4% specificity (area under the curve (AUC): 0.740, 95% CI: 0.711–0.768, *P* < 0.001).

**Conclusion:**

TRI is an independent predictor of CIN, and it may be used as a simple and reliable risk assessment of CIN in STEMI patients without the need for laboratory parameters.

## 1. Introduction

Contrast nephropathy (CIN) is characterized by an acute disruption in renal functions following exposure to contrast agents, and different studies have reported its incidence as 5–25% [1, 2]. Many factors are associated with the development of CIN, including advanced age, increased amount of contrast agent, basal renal failure, diabetes mellitus (DM), and hypertension (HT) [3]. It may develop more commonly after primary percutaneous intervention. Studies have shown that contrast nephropathy causes prolongation of hospitalization duration, increased in-hospital complications, and increased 1-year mortality [4, 5]. Early and correct recognition of CIN is crucial in the prevention of progression and improvement of outcomes [6].

The thrombolysis in myocardial infarction risk index (TRI) is a simple risk score designed for using at initial presentation to predict mortality in STEMI patients; it does not include any laboratory variables. The TRI is derived from three readily available clinical variables and is calculated using the equation (heart rate × (age/10) 2/systolic blood pressure) [7, 8]. TRI has been linked to mortality and morbidity in many cardiovascular diseases such as ST-elevation myocardial infarction (STEMI), pulmonary embolism, and acute heart failure [9].

Various laboratory parameters and scoring systems have shown to be successful at predicting the development of CIN [10]. In this study, we aimed to evaluate the effectiveness of TRI, which is a clinical score easy to calculate in STEMI patients undergoing primary percutaneous intervention.

## 2. Materials and Method

### 2.1. Study Population

A total of 963 patients who presented with the diagnosis of STEMI between September 2015 and January 2018 and underwent primary PCI (p-PCI) were retrospectively included. Patients with known allergy against contrast agents, those presented with cardiogenic shock, patients using oral anticoagulants, those with hematologic disease, chronic inflammatory or autoimmune disease, patients with a creatinine clearance < 60 mL/min, and patients with chronic renal failure who required dialysis were excluded from the study. Age, systolic blood pressure (SBP), and heart rate (HR) were obtained at the time of admission in all patients. For each patient, TRI score was calculated before PCI, and the relationship between TRI and the development of CIN was investigated. Patients' TRI was calculated using the formula “heart rate × (age/10) 2/SBP” [7]. Blood samples for the full blood count and the biochemistry parameters were obtained at the time of admission and 48 hours later from all patients. Written or verbal informed consents were received from all patients, and the study protocol was approved by the hospital's local ethics committee in accordance with the Helsinki Declaration and Good Clinical Practice Guidelines.

### 2.2. Definitions

STEMI was defined as the presence of ST elevation at least 1 mm in two or more continuous leads (2 mm for V1–V3) or new-onset left bundle branch block. All patients underwent p-PCI within the first 12 hours after the onset of the chest pain. Before PCI, all patients were given acetylsalicylic acid (ASA) (300 mg) and clopidogrel (600 mg) as well as unfractioned heparin (70 IU/kg IV). After the intervention, all patients were given 1 mg/kg of subcutaneous enoxaparin twice daily, 100 mg/day of ASA, and 75 mg/day of clopidogrel. Patients who were using antihypertensive agents or those have a systolic blood pressure, which was measured at rest with five minutes intervals in different time periods higher than 140 mmHg or a diastolic blood pressure higher than 90 mmHg, were considered hypertensive [11].

Patients who were using antidiabetics or having a postprandial blood glucose level higher than 200 mg/dL or fasting plasma glucose of at least two times above 126 mg/dL were considered diabetic [12].

Hyperlipidemia (HL) was defined as a total cholesterol higher than 200 mg/dL, triglycerides level higher than 160 mg/dL, and low-density lipoprotein level higher than 130 mg/dL [13]. Ejection fraction (EF) was calculated using the modified Simpson's method. For patients undergoing p-PCI, iopromide (Ultravist®) was used as the nonionic iso-osmolar contrast agent. All patients were hydrated (0.9% sodium chloride 1 mL/kg/hour) via intravenous route for 12 hours after the intervention. Blood samples were drawn before and 48 hours after p-PCI for measurement of serum creatinine. The creatinine clearance was calculated by using the Cockcroft–Gault formula: (140-age) ∗ (weight in kg) ∗ (0.85 if female)/(72 ∗ creatinine). CIN was defined as previously described and distinguished as grade 0 (serum creatinine increase <25% above baseline and <0.5 mg/dL above baseline), grade 1 (serum creatinine increase ≥25% above baseline and <0.5 mg/dL above baseline), or grade 2 (serum creatinine increase ≥0.5 mg/dL above baseline) [14]. Preexisting chronic kidney disease (CKD) was defined as having an estimated glomerular filtration rate (GFR) value <60 mL/min/1.73 m^2^.

### 2.3. Statistical Analysis

The data analysis was conducted using SPSS (version 20.0, SPSS Inc., Chicago, IL, USA) and MedCalc statistical software (trial version 12.7.8, Mariakerke, Belgium). Continuous variables are expressed as the mean ± standard deviation. Categorical variables were compared using Chi-square or Fisher's exact tests and summarized as percentages. The Kolmogorov–Smirnov test was used to evaluate the distribution of the continuous variables. To predict CIN, age, gender, DM, HT, HL, systolic and diastolic blood pressure, heart rate, history of prior myocardial infarction (MI), Killip ≥3, pre-MI medication, syntax score, TIMI flow, GFR, creatinine, contrast amount, and TRI were included in the univariate analysis. The parameters with *P* < 0.05 were included in the multiple logistic analysis. Receiver operating characteristic (ROC) curves were used to predict the future incidence of CIN.

## 3. Results

In this study, a total of 963 patients were included (mean age 58.1 + 11.9 and 77.3% male). A total of 128 (13.3%) patients developed CIN. Among these, 7.6% had grade 1 CIN and 5.7% had grade 2 CIN. The clinical characteristics as well as the angiographic and PCI features of the findings are listed in Tables 1–3.

Age, gender, DM, HT, SBP, diastolic blood pressure (DBP), heart rate, Killip >2, EF, basal creatinine, basal GFR, amount contrast agent, syntax score, TIMI flow, and TRI were statistically different in the CIN-positive group compared with the CIN-negative group.

Among the significant parameters in the univariate analysis (age, gender, DM, HT, heart rate, EF, GFR, TIMI flow, syntax score, contrast amount, and TRI), those that were also found to be significant in the multiple regression analysis included contrast amount (odds ratio (OR) = 1.010, 95% confidence interval (CI): 1.007–1.012, *P* < 0.001) and TRI ((OR) = 1.047, (CI): 1.020–1.075, *P* < 0.001) (Table 4).

**Figure 1:**
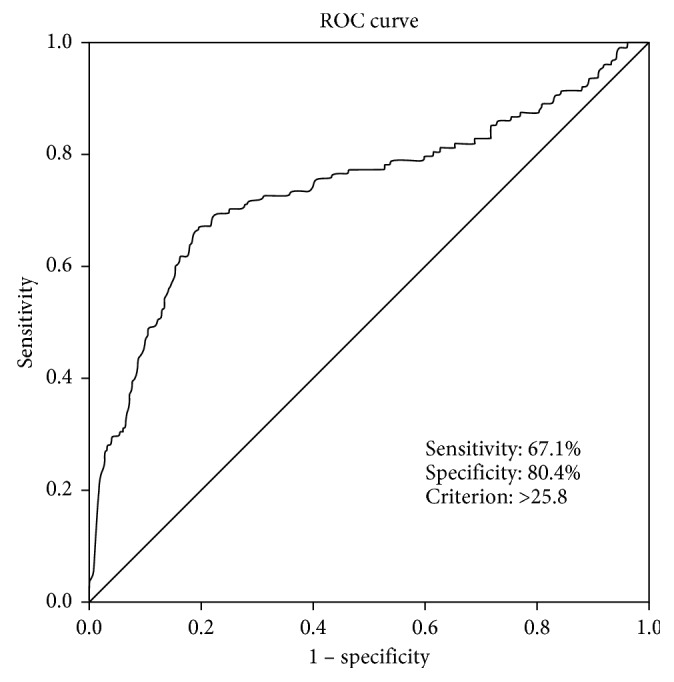
ROC curve analysis plot to determine the cut-off value of TRI in prediction of CIN.

The best TRI for CIN prediction was found to be 25.8 in the ROC curve analysis (AUC: 0.740, 95% CI: 0.711–0.768, *P* < 0.001). TRI ≥ 25.8 predicted CIN development with 67.1% sensitivity and 80.4% specificity (Figure 1).

## 4. Discussion

In our study, TRI was found to predict the development of contrast-induced nephropathy in STEMI patients who underwent PCI. It was found to be an independent predictor of CIN without the need for laboratory parameters.

CIN is a condition characterized by acute disruption in renal functions after exposure to contrast agents [15]. Medullary hypoxia due to renal artery vasoconstriction, direct cytotoxic effects of contrast agents, and decreased nitric oxide release secondary to the release of various mediators after the exposure are among the known pathophysiological mechanisms in the development of CIN [16]. In addition, protein precipitates due to inflammation secondary to renal medullary hypoxemia cause obstruction, playing a role in acute renal damage [17].

Previous studies have shown the association between the development of CIN and hospitalization duration, morbidity, and mortality [4]. Therefore, determination of the parameters that could predict the development of CIN before the intervention is of importance. Increased number of intervention because of the advancement in invasive cardiological techniques increases the risk for CIN [18]. Although various risk scores have been defined for prediction of the development of contrast-induced nephropathy after percutaneous coronary intervention (PCI), utility of these scores in clinical practice and their currency are limited [19, 20]. Especially, individual factors and characteristic variability of the populations on which the score systems are applied may have different effects on outcomes. Mehran's score is one of the most common risk scores in prediction of the risk for CIN [2]. In this scoring, a risk analysis is carried out with evaluation of many parameters. However, considering both numerical increase in the number of parameters and difficulties to take time for this in busy clinics, a risk score that can be applied more easily is warranted.

TIMI risk index is a score obtained by the formulation of age, systolic blood pressure, and heart rate, with a proven correlation with mortality and other outcome points in STEMI [7]. In a study, the relationship between TRI and long-term mortality and the development of heart failure in STEMI patients were demonstrated. In that study, although individual relationships of numerical values used in calculation of TRI (age, blood pressure, heart rate) were not showed, after the calculation this score was shown to be correlated with mortality [8]. Similarly, in our study, when separately evaluated age, systolic blood pressure, and heart rate were not significant predictors to indicate the development of CIN, while TRI was shown to independently predict the development of CIN. In many previous studies, various clinical factors have been shown to predict the development of CIN, although combined use of these parameters rather than individual use has been preferred for measurement of a score, and thus, their diagnostic value has been increased [21]. This suggests that better outcomes can be achieved by obtaining a few parameters using mathematical calculations. In a study, advanced age, anterior MI, and more amounts of contrast agent have been shown to be independently correlated with prediction of the development of CIN after p-PCI [22]. In another study, age has been shown as an independent predictor, although many studies have reported that SBP and heart rate were not independent predictors [9]. In our study, we found a significant correlation between the amount of contrast agents and development of CIN; age was not an independent predictor. In our study, it is not surprising to find a relationship between TRI, which contains pulse and systolic blood pressure that are the markers of hemodynamic changes and CIN. Previous studies have shown that disruption in renal blood flow causes acute renal damage with numerous neurohormonal mechanisms [5]. TRI provides ease of use with its features such as being a simple risk index used without a need for laboratory measurements and having only variables (age, heart rate, and systolic blood pressure).

The incidence of CIN has been reported to be higher compared with exposure to other elective contrast agents, because of the hemodynamic instability in primary percutaneous coronary intervention, the procedure itself, and the inability to take precautionary measure [4, 5]. Considering the relation of CIN development with morbidity and mortality, it is important to identify particularly high-risk patients for new strategies that can be developed in the treatment of CIN. Therefore, as is shown in our study, the use of TRI which is easy to calculate in prediction of the development of CIN could be useful in identification of the patient group which could be evaluated in the development of new treatment strategies.

## 5. Limitations

This is an observational, single-institution study, which had a relatively small sample size and was thus subject to various unaccounted confounders inherent in such an analysis. Our findings should be confirmed and the application of the risk score validated in a large multicenter trial. Our patient group included only STEMI patients, and other patients with acute coronary syndrome or stable angina were not included.

## 6. Conclusion

In this study, we showed that increased TRI is an easily applicable simple and useful score without laboratory parameters. TRI is an independent early predictor of CIN in STEMI patients who are undergoing coronary intervention. Early prediction may provide time to prevent the progression of CIN and improve its negative impacts on outcome.

## Figures and Tables

**Table 1:** Comparison of baseline clinical characteristics of patients with and without CIN after PCI.

Variables	CIN (−) (*n*=835)	CIN (+) (*n*=128)	*P* value
Age, years	56.8 ± 11.4	66.2 ± 12.3	<0.001
Age > 75	64 (7.7)	35 (27.3)	<0.001
Gender, male, (*n*, %)	660 (79.0)	84 (65.6)	0.001
Diabetes mellitus, (*n*, %)	175 (21)	51 (39.8)	<0.001
Hypertension, (*n*, %)	333 (39.9)	65 (50.8)	0.020
Smoking, (*n*, %)	328 (39.2)	45 (35.2)	0.191
Hyperlipidemia, (*n*, %)	332 (39.8)	43 (33.6)	0.182
Family history of CAD, (*n*, %)	193 (23.1)	32 (25)	0.725
Prior MI, (*n*, %)	89 (10.7)	12 (9.4)	0.659
Prior PCI, (*n*, %)	80 (9.6)	11 (8.6)	0.722
Prior CABG	27 (3.2)	3 (2.3)	0.589
Systolic blood pressure, mmHg	124 ± 41	131 ± 25	0.011
Diastolic blood pressure, mmHg	77 ± 14	81 ± 20	<0.001
Heart rate, (p/min)	76 ± 14	80 ± 20	<0.001
Killip class (≥II), (*n*, %)	104 (12.5)	43 (33.6)	<0.001
LV-EF, %	46.9 ± 7.7	43.2 ± 8.8	<0.001
TRI	32.9 ± 18.8	19.9 ± 9.9	<0.001

Values are expressed as mean ± SD or percentages. CAD, coronary artery disease; MI, myocardial infarction; PCI, percutaneous coronary intervention; CABG, coronary artery bypass graft; LV-EF, left ventricular ejection fraction; TRI, the thrombolysis in myocardial infarction risk index.

**Table 2:** Comparison of biochemical and hematologic variables of patients with and without CIN after PCI.

Variables	CIN (−) (*n*=835)	CIN (+) (*n*=128)	*P* value
Baseline creatinine, mg/dL	0.92 ± 0.35	1.09 ± 0.51	<0.001
72 h creatinine, mg/dL	0.99 ± 0.25	1.5 ± 0.79	<0.001
Δ-Cr	0.06 ± 0.02	0.41 ± 0.75	<0.001
Baseline GFR, mL/min/1.73 m^2^	88.6 ± 23.8	75.1 ± 31.0	<0.001
Glucose, mg/dL	150 ± 79	186 ± 102	<0.001
Hemoglobin, g/dL	13.2 ± 1.8	13.0 ± 2.0	0.156
CRP, mg/l	12.4 ± 10.4	17.7 ± 14.7	<0.001
LDL-cholesterol, mg/dL	115 ± 34	112 ± 37	0.328
HDL-cholesterol, mg/dL	38 ± 11	37 ± 11	0.523
Triglyceride, mg/dL	138 ± 84	128 ± 58	0.191

Δ-Cr, increase of creatinine in 72 hours; GFR, glomerular filtration rate; CK-MB, creatinine kinase myocardial band; CRP, C-reactive protein; LDL, low-density lipoprotein; HDL, high-density lipoprotein.

**Table 3:** Comparison of angiographic and treatment variables of patients with and without CIN after PCI.

Variables		CIN (−) (*n*=835)	CIN (+) (*n*=128)	*P* value
Amount of contrast media		232 ± 66	290 ± 89	<0.001
Previous medications, %				
Acetylsalicylic acid		13.8	9.4	0.171
Statin		19.4	8,5	0.037
ACE inhibitors/ARB		19.5	14.4	0.192
Beta-blocker		5	4.7	0.868
Calcium channel blocker		5	8	0.267
Oral antidiabetic		15	21.9	0.047
Insulin		5.7	21.1	<0.001
Infarct-related artery, (*n* %)	LM	3 (0.4)	1 (0.1)	0.155
	LAD	400 (47.9)	68 (53.1)	
	LCx	132 (15.8)	10 (7.8)	
	RCA	289 (34.6)	49 (38.3)	
	Other	11 (1.3)	—	
Syntax score, %		15.4 ± 6.8	16.8 ± 8.6	0.035
TIMI flow 3, (*n*, %)		775 (92)	101 (79)	<0.001

ACE, angiotensin-converting enzyme; ARB, angiotensin receptor blocker; LM, left main; LAD, left anterior descending artery; LCx, left circumflex artery; RCA, right coronary artery; TIMI, thrombolysis in myocardial infarction.

**Table 4:** Independent predictors of contrast-induced nephropathy in multivariate logistic regression analysis.

	Univariate analysis		Multivariate analysis		
	OR	*P* value	OR	95% CI	*P* value
Age	1.071	**<0.001**	1.025	0.996–1.055	
Sex	0.506	**<0.001**	1.156	0.686–1.948	
Diabetes mellitus	0.400	**<0.001**	0.700	0.437–1.122	
Hypertension	0.643	**0.020**	0.888	0.553–1.426	
Heart rate	1.024	**<0.001**	1.007	0.992–1.023	
LV-EF	0.945	**<0.001**	0.986	0.958–1.015	
TRI index	1.071	**<0.001**	1.047	1.020–1.075	**0.001**
GFR	0.978	**<0.001**	0.994	0.948–1.003	
TIMI flow	3.453	**<0.001**	1.556	0.830–2.918	
Syntax score	1.027	**0.035**	0.984	0.957–1.012	
Amount of contrast media	1.009	**<0.001**	1.010	1.007–1.012	**<0.001**

TRI, the thrombolysis in myocardial infarction risk index; TIMI, thrombolysis in myocardial infarction; LV-EF, left ventricular ejection fraction; GFR, glomerular filtration rate.

## Data Availability

Data can be requested on an individual case basis via the corresponding author.
